# A cholesterogenic gene signature for predicting the prognosis of young breast cancer patients

**DOI:** 10.7717/peerj.13922

**Published:** 2022-08-18

**Authors:** Xiaoping Li, Chaorong Zhou, Chaoran Qiu, Weiwen Li, Qihe Yu, Hui Huang, Yiwen Zhang, Xin Zhang, Liangliang Ren, Xin Huang, Qinghua Zhou

**Affiliations:** 1 Department of Breast Surgery, The First Affiliated Hospital of Jinan University, Guangzhou, China; 2 Department of Breast, Jiangmen Central Hospital, Jiangmen, China; 3 Department of Gastrointestinal Surgery, Jiangmen Central Hospital, Jiangmen, China; 4 Department of Oncology, Jiangmen Central Hospital, Jiangmen, China; 5 Department of Breast Surgery, Jiangmen Maternity & Child Health Care Hospital, Jiangmen, China; 6 Clinical Experimental Center, Jiangmen Key Laboratory of Clinical Biobanks and Translational Research, Jiangmen Central Hospital, Jiangmen, China; 7 Department of Breast Surgery, The First Affiliated Hospital of Jinan University; The Biomedical Translational Research Institute, Faculty of Medical Science, Jinan University, Guangzhou, China

**Keywords:** Breast cancer, Differentially expressed genes, Prognostic model, Cholesterogenic genes, Lipid metabolism

## Abstract

**Purpose:**

We aimed to establish a cholesterogenic gene signature to predict the prognosis of young breast cancer (BC) patients and then verified it using cell line experiments.

**Methods:**

In the bioinformatic section, transcriptional data and corresponding clinical data of young BC patients (age ≤ 45 years) were downloaded from The Cancer Genome Atlas (TCGA) database for training set. Differentially expressed genes (DEGs) were compared between tumour tissue (*n* = 183) and normal tissue (*n* = 30). By using univariate Cox regression and multi COX regression, a five-cholesterogenic-gene signature was established to predict prognosis. Subgroup analysis and external validations of GSE131769 from the Gene Expression Omnibus (GEO) were performed to verify the signature. Subsequently, in experiment part, cell experiments were performed to further verify the biological roles of the five cholesterogenic genes in BC.

**Results:**

In the bioinformatic section, a total of 97 upregulated genes and 124 downregulated cholesterogenic genes were screened as DEGs in the TCGA for training the model. A risk scoring signature contained five cholesterogenic genes (risk score = −1.169 × GRAMD1C −0.992 × NFKBIA + 0.432 × INHBA + 0.261 × CD24 −0.839 × ACSS2) was established, which could differentiate the prognosis of young BC patients between high-risk and low-risk group (<0.001). The prediction value of chelesterogenic gene signature in excellent with AUC was 0.810 in TCGA dataset. Then the prediction value of the signature was verified in GSE131769 with *P* = 0.033. In experiment part, although the downregulation of CD24, GRAMD1C and ACSS2 did not significantly affect cell viability, NFKBIA downregulation promoted the viability, colony forming ability and invasion capability of BC cells, while INHBA downregulation had the opposite effects.

**Conclusion:**

The five-cholesterogenic-gene signature had independent prognostic value and robust reliability in predicting the prognosis of young BC patients. The cell experiment results suggested that NFKBIA played a protective role, while INHBA played the pro-cancer role in breast cancer.

## Introduction

Increased working pressure and unhealthy lifestyles have led to the rapid growth of breast cancer (BC) in women. In the United States, approximately 11% of the 230,000 new cases of invasive BC were diagnosed annually in women aged 45 years or younger (Rosenberg, Newman & Partridge, 2015). In Asia, young BC patients account for a much higher proportion than that in the US, which is up to 20% (Ribnikar et al., 2017). Younger BC patients have more risk factors than older BC patients, including a higher proportion of hormone receptor-negative, high-grade, and HER2-positive tumours (Azim Jr & Partridge, 2014). Hence, understanding the intrinsic mechanism is vital for selecting the proper treatment for young BC patients.

Metabolic reprogramming or cancer metabolism is the hallmark of malignant tumours, which indicates a share of pathways observed in highly proliferative tumours and cancer cells (Tang et al., 2018). In recent years, an increasing number of scientists have focused their research on the cancer metabolic field to further uncover the mechanism of tumorigenesis (Tang et al., 2021). Regarding BC, Mehla & Singh (2020) demonstrated that the cholesterogenic subtype is correlated with better outcomes, potentially due to more energy expenditure. Leignadier et al. (2017) highlighted that the inhibition of the cholesterogenic activity of the microsomal antiestrogen binding site (AEBS) could lead to tamoxifen resistance in BC patients. Some new drugs targeting tumour metabolism have been developed, but none of them have been shown to be effective (Akhenblit & Pagel, 2016). Alterations in cellular metabolic pathways of lipid and cholesterol synthesis have been linked to tumorigenesis and cancer progression but have not been utilized in clinical diagnosis (Pampalakis et al., 2015). The establishment of a cholesterogenic gene signature to predict the prognosis of younger BC patients is promising.

In this study, we retrospectively reviewed a cohort of BC patients aged 45 years or younger in The Cancer Genome Atlas (TCGA) database. By analysing the correlation between all cholesterogenic RNAs and the survival status of 183 patients, we established a five-cholesterogenic-gene signature and then validated it in a Gene Expression Omnibus (GEO) dataset (GSE131769). Then the function of the five cholesterogenic genes were verified in cell line experiment.

## Materials and Methods

### Bioinformatic part

#### TCGA (BRCA-US) and GEO (GSE131769) data collection

BC transcriptome data (HTSeq-FPKM) and clinical materials were downloaded from the TCGA (https://cancergenome.nih.gov) and GEO database (https://www.ncbi.nlm.nih.gov/geo). The BC patients from the TCGA were from the United State National Cancer Institute. While the patients from GSE131769 (GEO database) were collected from Yonsei University College of Medicine, Korea. The samples from these two datasets did not overlap. The selection criterion was age ≤45 years. A total of 183 tumour samples and 30 normal samples in the TCGA were included as the training set. The GSE131769 dataset, with data from the GPL6947 Illumina HumanHT−12 V3.0 expression bead chip, was used for clinical external validation. After excluding patients older than 45 years, 130 patients with expression array data and survival data were enrolled.

#### Cholesterogenic gene preparation and differentially expressed genes (DEGs)

Cholesterogenic genes were downloaded from the Molecular Signatures Database (MSigDB) (Liberzon et al., 2011). Cholesterogenic gene sets were considered if the description contained “cholesterol”. A total of 30 cholesterol-related gene sets were selected (Table S1).

The transcriptional expression of these cholesterogenic genes was incorporated into the TCGA samples using Perl (Perl is a highly capable, feature-rich programming language. The Perl package was uploaded to the sorce code). Then, we obtained the DEGs by comparing the expression of the RNA sequences of 183 tumour samples with those of 30 normal samples using R language, where the “limma” and “ggplot2” packages were applied for statistical analysis (“limma” package was used for differential analysis; “ggplot2” package was used for drawing volcano plot). In order to include all the possible genes in the preliminary screening, we just use *P* < 0.1 as the cut-off value.

#### Functional and pathway enrichment analysis

Database for Annotation, Visualization and Integrated Discovery (DAVID) version 6.8 (https://david.ncifcrf.gov/summary.jsp) was used to analyse the Gene Ontology (GO) functional enrichment and Kyoto Encyclopedia (KEGG) pathway enrichment of the DEGs (Jiao et al., 2012). The description of GO functional enrichment consisted of biological processes (BPs), molecular functions (MFs), and cellular components (CCs). R language was employed for visualization using the “ClusterProfiler”, “enrichplot” and “ggplot2” packages. The cut-off values were set at *P* < 0.05 and false discovery rate (FDR) < 0.05.

#### Construction of a five-gene signature in the TCGA dataset

First, we used the “survival” package in R to carry out univariate Cox regression to identify prognosis-related cholesterogenic genes. Then, by combining survival status and survival time of the patients, multi COX regression was performed to further screen out survival-related genes with corresponding hazard ratio value and correlation coefficient. “Survival” package in R was applied. Third, the genes closely correlated with survival were used to construct a risk scoring model. After filtering out 4 patients with incomplete follow-up data, we stratified 179 patients into high-risk and low-risk groups according to the median risk score and compared the overall survival between the two groups with Kaplan–Meier analysis using the “survminer” package. Fourth, univariate and multivariate analyses were implemented to assess the prognostic value of the model based on age, clinical stage, surgical method, and risk score. Fifth, the receiver operating characteristic (ROC) curve was drawn to evaluate the predictive value of the signature using the “survival ROC” package.

#### Subgroup analysis in TCGA and external validation in GSE131769

Subgroup analysis was carried out according to the clinical features of the patients in the TCGA database. The patients were divided into two groups by the dichotomy method according to age (≤40 years and >40 years), surgical method (breast-conserving surgery (BCS) and mastectomy), T stage (T1-2 and T3-4) and N stage (N0-1 and N2-3) to verify the predictive ability of the signature.

BC patients aged 45 years or younger in GSE131769 with microarray data and corresponding clinical information were used for further external validation.

#### Expression of the five screened cholesterogenic genes in normal tissues and tumour tissues

The expression of GRAMD1C, NFKBIA, INHBA, CD24, and ACSS2 in the TCGA training set was subjected to log2 transformation. We compared the expression of the above five cholesterogenic genes between normal tissues and tumour tissues. The *P* value was set at 0.05.

### Experimental part

#### Cell culture and transient transfection

The human BC cell lines MDA-MB-231 and MCF7 were obtained from the American Type Culture Collection (Manassas, VA, USA). The cells were cultured in Dulbecco’s modified Eagle medium (DMEM, Gibco, USA) with 10% foetal bovine serum (FBS, Gibco, USA) and incubated at 37 °C in a humidified atmosphere with 5% CO_2_.

Cells were digested with trypsin and seeded in 24-well plates at a density of 7 × 10^4^ cells/500 µL per well. Transient transfection of the plasmids was performed using Lipofectamine 3000 reagent (Invitrogen, Waltham, MA, USA) according to the manufacturer’s instructions.

#### Cell counting kit-8 (CCK-8) analysis, colony formation assay and transwell migration assay

For CCK-8 analysis, the cells (2 ×10^3^) were seeded into 96-well plates. At the indicated time point, 100 µL of CCK-8 dye solution was added to each well and incubated for 1 h, and the absorbance was measured at a wavelength of 450 nm. Cell viability was calculated by the following formula: cell viability = (optical density (OD) value of the experimental group/OD value of the control group) × 100%. For the colony formation assay, the indicated cells (0. 2 ×10^3^) were seeded into six-well plates and maintained at 37 °C. After 10 days, colonies were fixed with 10% formaldehyde for 15 min and stained with 1.0% crystal violet for 30 s. Colony morphologies were captured by a light microscope (Olympus).

Matrigel (BD Biosciences, Bedford, MA, USA) was prebalanced at 4 °C and mixed with 8 times the volume of precooled blank culture medium. An 80 µL Matrigel-medium mixture was coated on the upper polycarbonate 24-well culture chamber. Cells (1 ×10^4^) were plated on the top side coated with Matrigel and incubated at 37 °C for 22 h, followed by removal of the cells inside the upper chamber using cotton swabs. Invaded cells remaining on the lower membrane surface were fixed in 1% paraformaldehyde, stained with crystal violet, and counted (ten random fields per well, 100 × magnification).

### Statistical analysis

The data are presented as the mean ± standard deviation (SD). Statistical analysis of the results was conducted by one-way analysis of variance (ANOVA) or unpaired two-tailed Student’s *t* test, as appropriate. All statistical analyses were performed using GraphPad Prism 5.0 software, Perl language and R language.

## Results

### Bioinformatic part

#### Differentially expressed cholesterogenic genes and enrichment analysis

We downloaded TCGA-BRCA RNA-Seq data (HTSeq-FPKM) and clinical features from the TCGA database, which contained 1098 tumour samples and 123 normal samples. We selected patients aged 45 years or younger. Finally, 183 tumours and 30 normal tissues were included.

Then, cholesterol-related genes were extracted from MSigDB, with a total of 345 cholesterogenic genes (Table S2). We compared the expression of cholesterogenic genes in tumour tissues and normal tissues to obtain the DEGs. Considering only primary filtering, *P* = 0.1 was set as the criterion. A total of 97 upregulated genes and 124 downregulated genes were screened out (Fig. S1).

**Figure 1:**
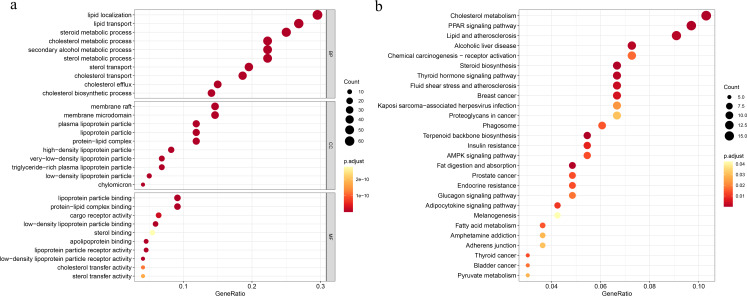
Functional and pathway enrichment analyses. (A) Functional enrichment analysis, BP, biological process; CC, cellular component, MF, molecular function; (B) KEGG pathway.

To explore the underlying mechanisms of the DEGs, we conducted GO functional and KEGG pathway enrichment analyses by using DAVID 6.8. In BP analysis, the lipid localization ranked the first. The membrane raf, membrane microdomain and membrane region were enriched in CC analysis. MF analysis showed that the DEGs were mainly enriched in lipoprotein particle binding and protein-lipid complex binding, which play equally important roles. Moreover, cholesterol metabolism was the most significant pathway in the KEGG analysis (Fig. 1).

#### Construction of the cholesterogenic gene signature in the TCGA-BRCA dataset and survival analysis

**Figure 2:**
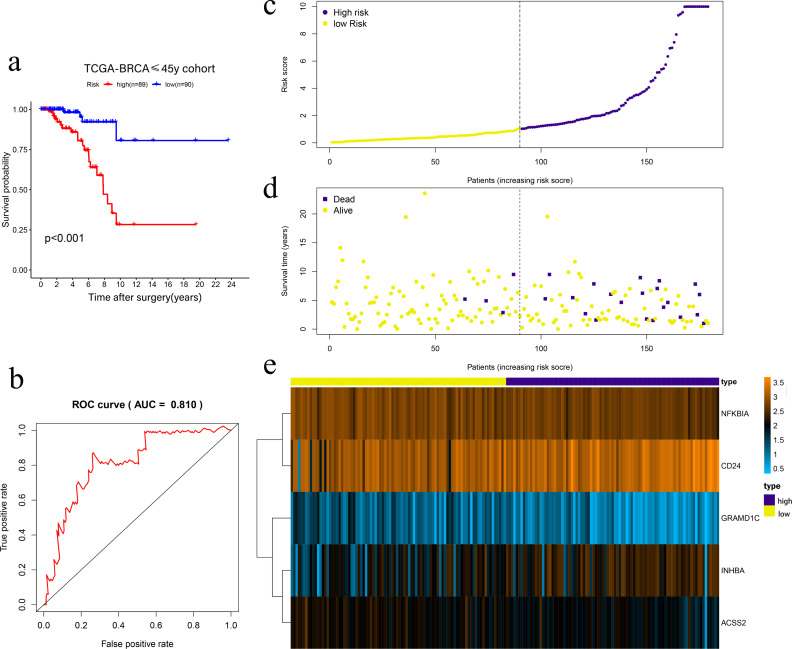
Risk scoring gene signature of five cholesterogenic genes in 179 ≤45-year-old breast cancer patients from the TCGA. (A) Survival analysis of the low-risk and high-risk groups; (b) ROC curve of the risk scoring signature; (c) Risk score distribution; (D) Patient survival status distribution; (E) Heatmap of five cholesterogenic genes in the high-risk and low-risk groups.

Univariate Cox regression was conducted to filter the survival-related cholesterogenic genes. GRAMD1C, NFKBIA, INHBA, CD24, MYLIP, ACSS2, and NR1H3 were screened out with the criteria of *P* < 0.05. Multi COX regression was performed to further select prognosis-related genes (GRAMD1C, NFKBIA, INHBA, CD24, and ACSS2). We constructed a five-gene signature in the TCGA cohort, with risk score formula = ∑^i^ (coefi*expri), (with i meant the number of genes, coefi meant the coefficiency of the genes, and expri meant the genes in the signature). As a result, Risk scores were calculated according to the following formula: risk score = − 1.169 × GRAMD1C − 0.992 × NFKBIA + 0.432 × INHBA + 0.261 × CD24 − 0.839 × ACSS2. Four patients with follow up <3 months were excluded, the remaining 179 patients were divided into the high-risk group (*n* = 89) and the low-risk group (*n* = 90) according to the median risk score. Kaplan–Meier survival analysis showed that the low-risk group had a significantly better prognosis than the high-risk group (*P* < 0.001) (Figs. 2A, 2C, 2D, 2E). The ROC curve showed that the area under the curve (AUC) value was 0.810 in TCGA dataset, which implied that the five-gene signature had excellent power in predicting the survival of young BC patients (Fig. 2B).

**Figure 3:**
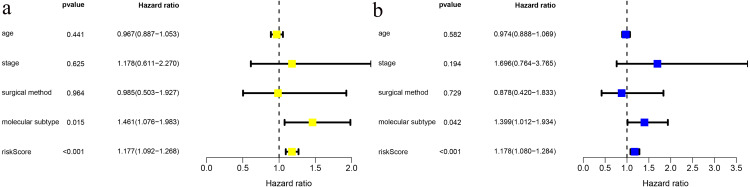
Risk factor analysis of ≤ 45-year-old breast cancer patients from the TCGA demonstrates that the risk scoring signature is an independent risk factor. (A) Univariate Cox regression result; (B) Multivariate Cox regression result.

We combined age, clinical stage, surgical method (BCS vs. mastectomy), T stage, N stage and risk score in univariate and multivariate factor analyses. We found that the risk score was an independent risk factor for prognosis in young BC patients (Fig. 3).

#### Subgroup analysis in TCGA and external validation in GSE131769

**Figure 4:**
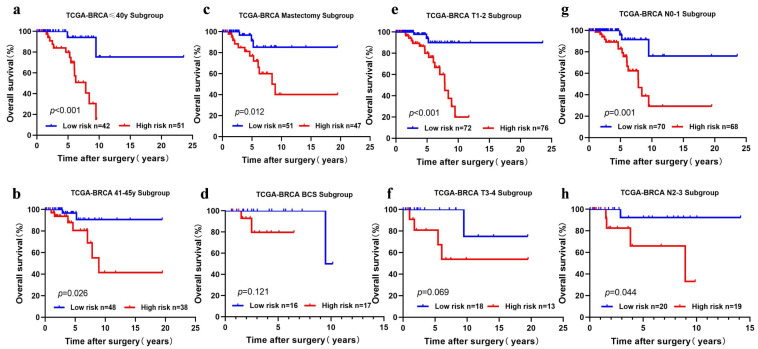
Risk scoring gene signature of five cholesterogenic genes in different clinical subgroups of the TCGA cohort. (A, B) The ≤ 40-year and 41-45-year subgroups; (C, D) The mastectomy and breast-conserving surgery subgroups; (E, F) The T1-2 and T3-4 subgroups; (G, H) The N0-1 and N2-3 subgroups.

We performed subgroup analysis by stratifying the young BC patients in TCGA-BRCA according to clinical features (age, T stage, N stage, and surgical method). The five-cholesterogenic-gene signature could be applied to differentiate prognosis in different subgroups of patients, including the age ≤40 years and age>40 years subgroups, the T1-2 subgroup, the N0-1 and N2-3 subgroups, and the mastectomy subgroup (*P* < 0.05) (Figs. 4A, 4B, 4C, 4E, 4G, 4H). We noticed a different survival trend in the BCS and T3-4 subgroups, although the *P* value was above 0.05 (Figs. 4D, 4F). This result is likely because of the small sample sizes of these subgroups.

**Figure 5:**
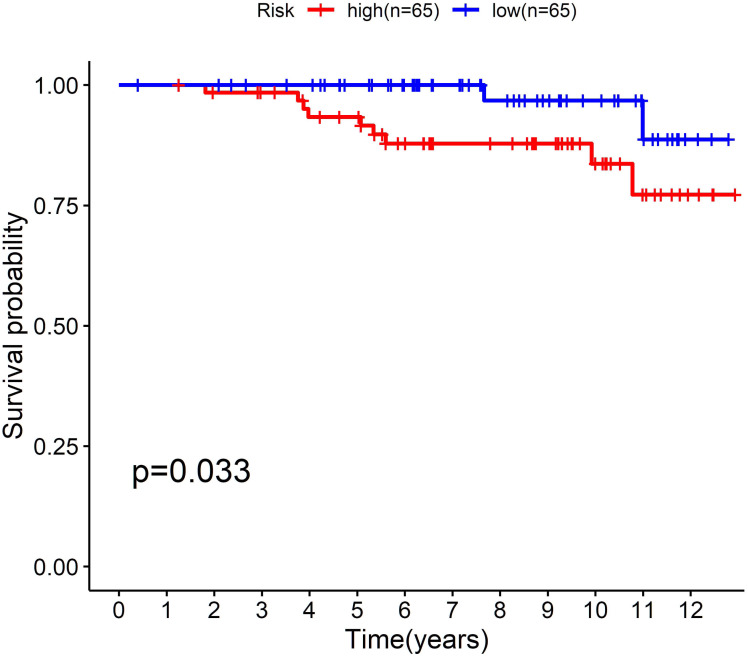
External validation of the five-cholesterogenic-gene signature in GSE133769. 130 young breast cancer patients were divided into high risk and low risk group by median risk score. The low risk group showed better survival outcome than high risk group (*P* = 0.033).

To further confirm the model, we performed external validation by using GSE131769, which contains 130 patients aged 45 years or younger. By calculating the risk score for each person, we found that the cholesterogenic gene signature also worked well in the external validation set. The low-risk group (*n* = 65) had a much more favourable prognosis than the high-risk group (*n* = 65, *P* = 0.033) (Fig. 5).

#### Expression of the five cholesterogenic genes in normal tissues and tumour tissues

The expression of CD24 and INHBA was significantly high in tumour tissue (*P* = 0.0102 and *P* < 0.001). The expression of GRAMD1C, NFKBIA, and ACSS2 was significantly higher in normal tissues than in tumour tissues (*P* < 0.001, *P* = 0.0096, and *P* < 0.001, respectively), which indicated that CD24 and INHBA were risk genes and that GRAMD1C, NFKBIA, and ACSS2 were protective genes (Fig. S2).

### Experiment part: inhibiting the proliferation and invasion ability of BC cells, NFKBIA plays an anti-carcinogenic effect, while INHBA plays the opposite role

**Figure 6:**
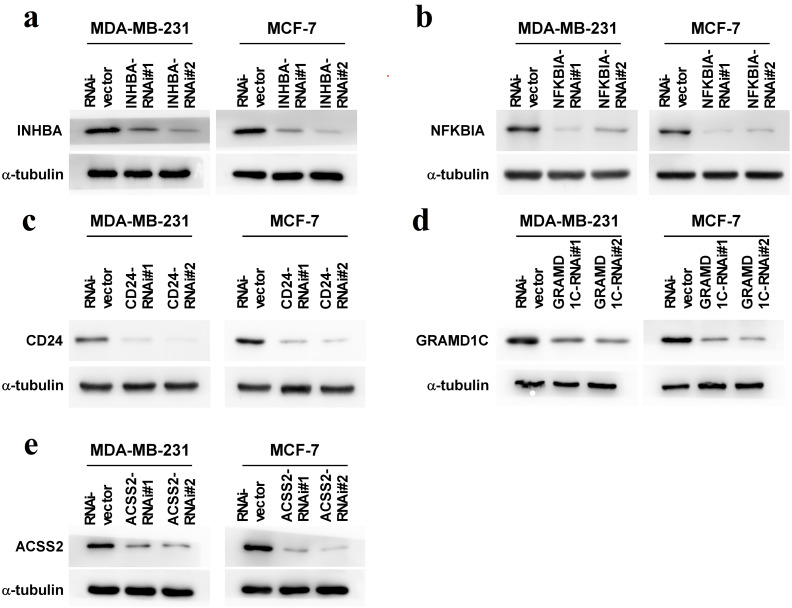
(A–E) Expression levels of INHBA, CD24, GRAMD1C, NFKBIA, and ACSS2 in MDA-MB-231 and MCF7 breast cancer cells were reduced by siRNA treatment.

**Figure 7:**
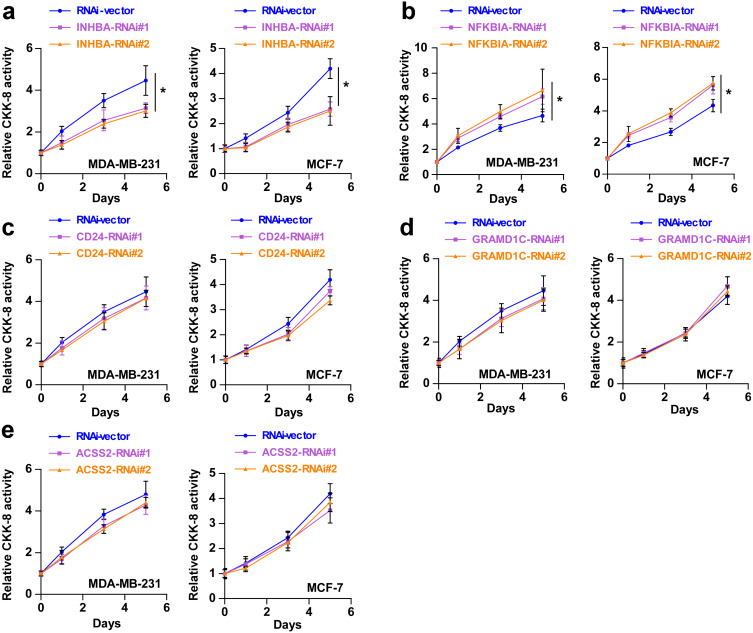
(A–E) After treatment with various siRNAs, including siRNAs against INHBA, CD24, GRAMD1C, NFKBIA, and ACSS2, the viability of MDA-MB-231 and MCF7 breast cancer cells was evaluated by CCK-8 assay. The data are represented as the mean ± SD (*n* = 5). * *p* < 0.05 vs. the control group.

**Figure 8:**
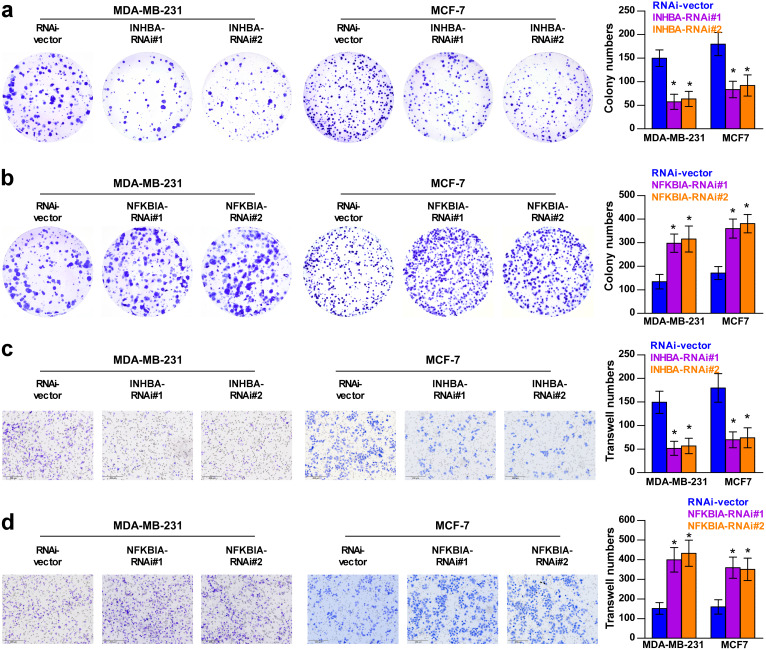
Colony forming ability (A, B) and invasion capability (C, D) of breast cancer cells were dramatically weakened with INHBA siRNA intervention but significantly increased by NFKBIA siRNA intervention. The data are represented as the mean ± SD (*n* = 5), * *p* < 0.05 vs. the control group.

To explore the biological roles of the five cholesterogenic genes in BC, we silenced gene expression by siRNA in MDA-MB-231 and MCF7 BC cells. We successfully inhibited the expression levels of these five cholesterogenic genes in BC cells by siRNA (Fig. 6). The results of the CCK-8 assay showed that the downregulation of INHBA inhibited the viability of BC cells, and the downregulation of NFKBIA promoted the viability of BC cells, while the downregulation of CD24, GRAMD1C and ACSS2 had no significant effect on cell viability (Fig. 7). Then, we further explored the effects of these two genes on the colony forming ability and the invasion capability of BC cells. The results showed that the colony forming ability and the invasion capability of BC cells were also significantly impaired by silencing INHBA, while the downregulation of NFKBIA enhanced these abilities (Fig. 8).

## Discussion

Cell proliferation requires nutrition, energy and biosynthetic activity to replicate all macromolecular components in each step of the cell cycle (DeBerardinis et al., 2008). Increasing reports have demonstrated that lipid metabolism is highly reprogrammed in cancer (Abramson, 2011; Santos & Schulze, 2012; Schulze & Harris, 2012). Lipid synthesis is significantly upregulated to meet the demand for increased membrane biosynthesis (Guo, Bell & Chakravarti, 2013; Menendez & Lupu, 2007; Yoon et al., 2007), and lipid uptake and storage are also upregulated in cancer (Accioly et al., 2008; Zhao et al., 2017). Interestingly, previous evidence showed that cholesterol, together with its metabolites and precursors, regulated tumorigenesis and promoted biological processes, such as the oncogene driver pathway, ferroptosis, and tumor microenvironment differentiation (Xu et al., 2020). In this respect, cholesterol inhibitors, including statins, are utilized in tumor therapies (Bathaie et al., 2017). Notably, some studies have demonstrated that metabolite 27-hydroxycholesterol (27HC) can serve as an oestrogen, increasing the proliferation of oestrogen receptor-positive BC cells. They further suggested that breast tumours displayed multiple levels of dysregulated cholesterol homeostasis and metabolism, all of which can be used to develop therapeutic strategies (Nelson, 2018; Nelson, Chang & McDonnell, 2014). Therefore, we hypothesized that cholesterol-related genes could be prognostic indicators of BC.

In this study, based on TCGA and GEO data, we identified a total of 221 cholesterogenic genes (DEGs) in young BC patients, which included 97 upregulated genes and 124 downregulated genes. To further explore the main underlying mechanisms of the DEGs, the GO enrichment analysis revealed that genes mainly involved lipid localization, membrane raf, membrane microdomain, membrane region, lipoprotein particle binding and protein-lipid complex binding. The KEGG pathway enrichment analysis revealed that cholesterol metabolism was the most significant pathway. These results strongly indicate that cholesterol, lipids and their metabolism are significantly influencing factors of the occurrence and development of BC. A previous study showed that low-density lipoprotein cholesterol can promote the proliferation, metastasis, loss of attachment function and transformation from endothelial cells to mesenchymal cells of BC cells. They also showed that cells exposed to low-density lipoprotein cholesterol activated the ErbB2 pathway and reduced the expression of adhesion molecules, which contribute to the proliferation and metastasis of BC (dos Santos et al., 2014).

Therefore, we hypothesized that establishing a cholesterogenic gene signature could predict the prognosis of young women with BC. In the present study, univariate Cox and multi COX regression analyses were performed to identify prognosis-related cholesterogenic genes. Finally, a prediction signature was constructed. The signature could differentiate the risk of young BC patients well, with an AUC value of 0.810. In addition, we confirmed that the risk score was an independent risk factor for prognosis in young BC patients. Moreover, the signature was validated in the GSE131769 validation set, which suggested that it was a robust model.

Among these five prognosis-related genes, we found that INHBA and CD24 were oncogenic genes, while GRAMD1C, NFKBIA and ACSS2 were tumour suppressor genes. To further verify the specific effects of these cholesterogenic genes on the proliferation viability and invasion ability of BC cells, we silenced the expression of these genes by siRNA in MDA-MB-231 and MCF7 cells. Interestingly, in our study, downregulation of INHBA decreased the viability, colony forming ability and invasion capability of BC cells, while downregulation of NFBKIA showed the opposite results. The conclusion of the cell line experiments was consistent with the prediction model. However, the downregulation of CD24, GRAMD1C and ACSS2 did not significantly affect cell viability. Not surprisingly, it is well known that the occurrence and development of cancer is a complex process of multiple gene—environment interactions, and the variation in a single gene may not cause the entire change in cancer cells.

Previously, a study reported that the downregulation of GRAMD1C could be a promising predictor of poor prognosis in renal clear cell carcinoma (Hao et al., 2019). GRAM domain containing 1C (GRAMD1C), an uncharacterized protein belonging to the GRAM domain family of proteins, is a cholesterol transporter that mediates the non-vesicular transport of cholesterol from the plasma membrane to the endoplasmic reticulum (Clark et al., 2003). The above finding was consistent with our findings. Our study was the first to report the prognostic value of GRAMD1C in BC. NFKBIA, also known as nuclear I *κ*B *α*, was a component of the NF- *κ*B pathway, which was involved in the regulation of several tumours (Kim et al., 2016; Weniger & Küppers, 2016). Weng et al. (2018) reported that NFKBIA can suppress colon tumours by directly targeting piR-1245. Our study also confirmed the protective function of NFKBIA in BC, but the potential mechanism needed further exploration. Acetyl-CoA synthetase 2 (ACSS2) was upregulated during metabolic stress, which promotes the uptake and utilization of acetate for AcCoA synthesis in stressed cancer cells (Schug et al., 2015). Increasing studies had shown that the genetic depletion of ACSS2 inhibits tumour growth in a wide variety of cancers (Chen et al., 2017; Chen et al., 2015). Pharmacological inhibition of ACSS2 as a single agent impaired breast tumour growth (Miller et al., 2021). ACSS2 served as an anticancer agent for BC cells by targeting cancer metabolism genes (Comerford et al., 2014). ACSS2 played a protective role in our model, although silencing ACSS2 did not change BC cell viability. INHBA and CD24 were risk factors among young BC patients. Inhibin *β*A (INHBA) was a member of the transforming growth factor- *β* superfamily, which was involved in the invasion and metastasis of a variety of malignant tumours (Okano et al., 2013; Seder et al., 2009). Kalli et al. (2019) indicated that knocking down INHBA levels in BC cells delayed the growth of primary tumours by suppressing migration *in vitro* and inhibiting the formation of lung metastases *in vivo*. In this study, we confirmed that knockdown of INHBA expression by siRNA inhibited the cell viability, weakened the colony forming ability and suppressed the invasion capability of BC cells. However, the primary mechanisms of the INHBA-mediated promotion of BC cell activity remained elusive. Further studies are warranted to examine the potential involvement of the pathway in the INHBA-mediated promotion of BC cell activity. CD24 was a small sialoglycoprotein that is associated with the development, invasion, and metastasis of cancer cells (Tarhriz et al., 2019). In this prognostic signature, we found that CD24 was a cancer-promoting factor. Mansoori et al. (2019) revealed that BC samples with advanced histological grade showed higher CD24 expression. Based on the studies above, our findings indicated that INHBA and CD24 were indicators of poor prognosis in young BC patients.

A novelty this research was the screening out the five prognostic-related cholesterogenic genes and constructing the signature which could predict the outcome of young breast cancer patients. By searching Pubmed, we found this was the first study focus on prognosis of young BC patients by cholesterogenic genes signature. Also the five cholesterogenic genes were further validated in experiment which could let us have a deeper understanding of the function of the genes.

However, there were a few limitations in our study. First, the clinical data were retrieved from the TCGA, which lacked some important information, including data on recurrence, endocrine therapy, and chemotherapy details. A large cohort prospective study is needed to further confirm this conclusion. Second, young BC patients were a heterogeneous group, which contained the luminal A, luminal B, triple-negative and HER-2 subtypes. Numerous patients lacked such data, which limited us from further studying the relationship between the molecular subtype and the signature. Third, the intrinsic mechanisms of the five cholesterogenic genes need to be further clarified *in vitro* experiments.

## Conclusions

In summary, based on bioinformatics, we successfully screened five prognostic-related cholesterogenic genes, and for the first time constructed a prognostic prediction model based on cholesterogenic genes in TCGA dataset and then verified in GSE131769 dataset. Through the cell experiment results, we found that NFKBIA played a protective role, while INHBA played the pro-cancer role. The present study provided a promising approach for predicting the prognosis of young BC patients.

##  Supplemental Information

10.7717/peerj.13922/supp-1 Supplemental Information 1 Differentially expressed cholesterogenic genes between tumour and normal tissues in the TCGA ≤ 45-year-old breast cancer cohort Green symbols represent downregulated genes, and red symbols represent upregulated genes.

10.7717/peerj.13922/supp-2 Supplemental Information 2 Protein–protein interactions (PPIs) of differentially expressed cholesterogenic genes

10.7717/peerj.13922/supp-3 Supplemental Information 3 Expression of five cholesterogenic genes in tumour and normal tissues (A) GRAMD1C; (B) NFKBIA; (C) INHBA; (D) CD24; (E) ACSS2. The data are represented as the mean ±SD. * *P* = 0.0096, ** *p* = 0.0102 and *** *p* < 0.001.

10.7717/peerj.13922/supp-4 Supplemental Information 4 Raw data

10.7717/peerj.13922/supp-5 Supplemental Information 5 Cholesterol related gene set in GSEA

10.7717/peerj.13922/supp-6 Supplemental Information 6 The cholesterogenic genes expression of 183 tumors and 30 normal tissues

## Additional Information and Declarations

### DNA Deposition

The following information was supplied regarding the deposition of DNA sequences: The BC transcriptome data (HTSeq-FPKM) and clinical materials are available at the TCGA (https://cancergenome.nih.gov, search term: “TCGA-BRCA”) and GEO: GSE131769. The cholesterogenic genes are available at the Molecular Signatures Database (MSigDB), search term: “cholesterol”.

### Data Availability

The following information was supplied regarding data availability: The raw data are available in the Supplementary File.
