# Supplementary figures and images for: A cholesterogenic gene signature for predicting the prognosis of young breast cancer patients
Source: PeerJ. 2022 Aug 18;10:e13922. doi: 10.7717/peerj.13922 (PMC9393010; doi:10.7717/peerj.13922)

# Volcano

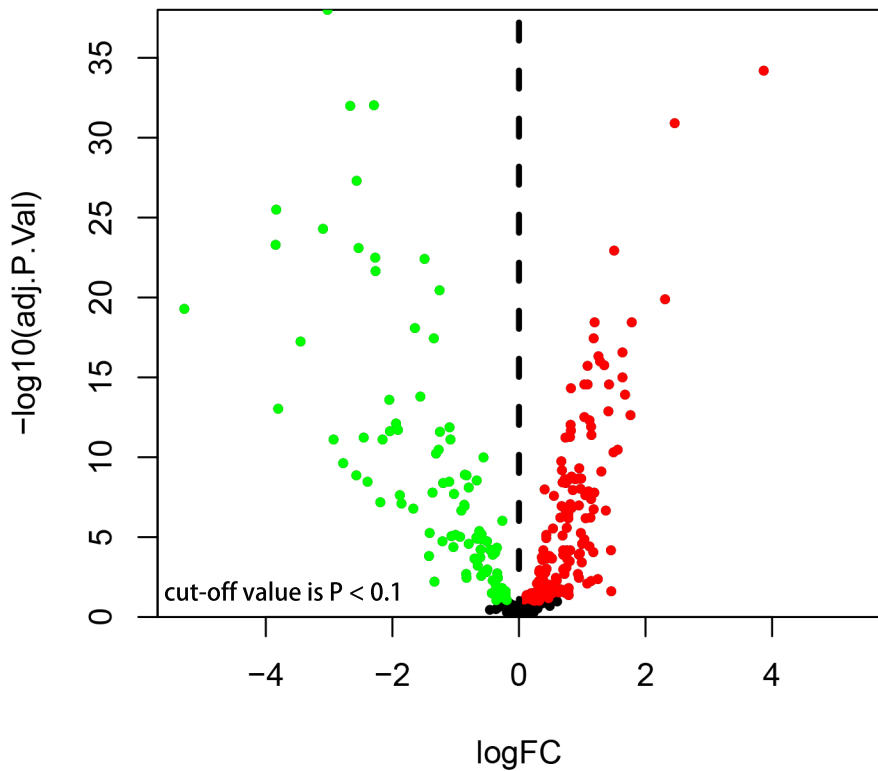

Supplement: Supplemental Information 1 — Green symbols represent downregulated genes, and red symbols represent upregulated genes. [file peerj-10-13922-s001.pdf]

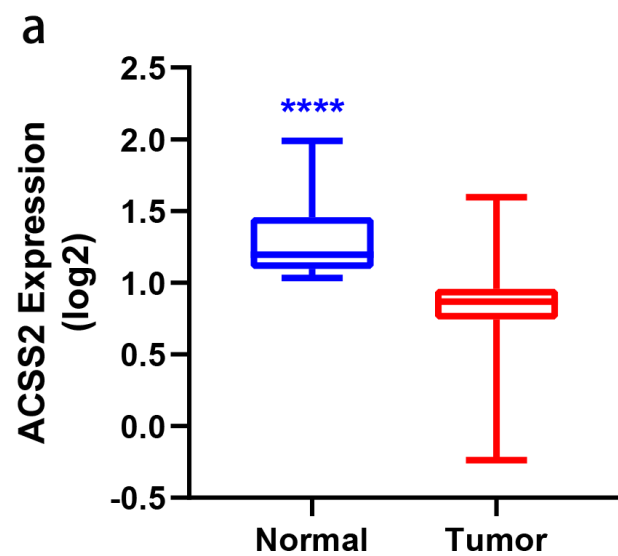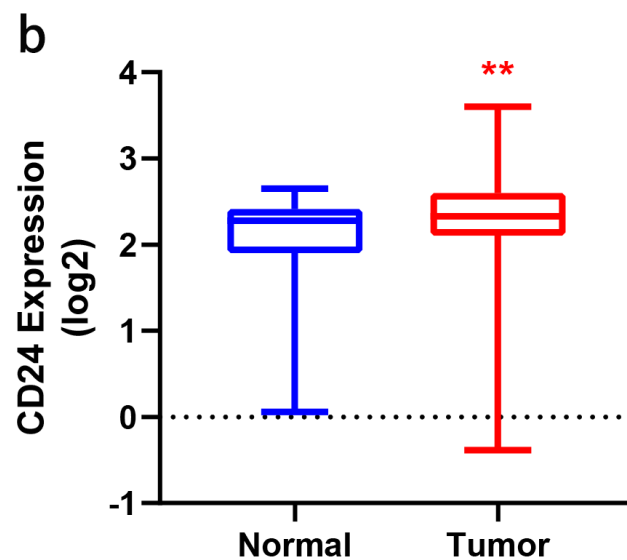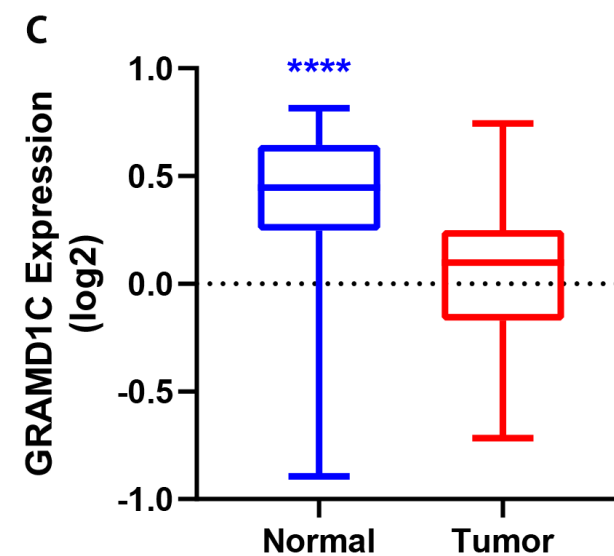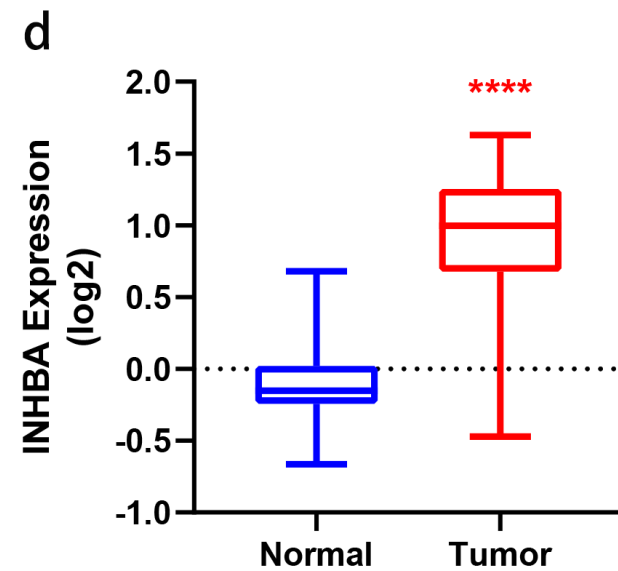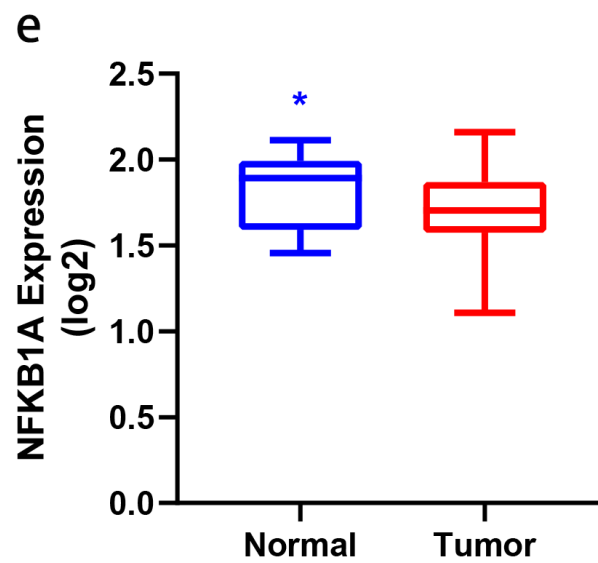

Supplement: Supplemental Information 2 [file peerj-10-13922-s002.pdf]

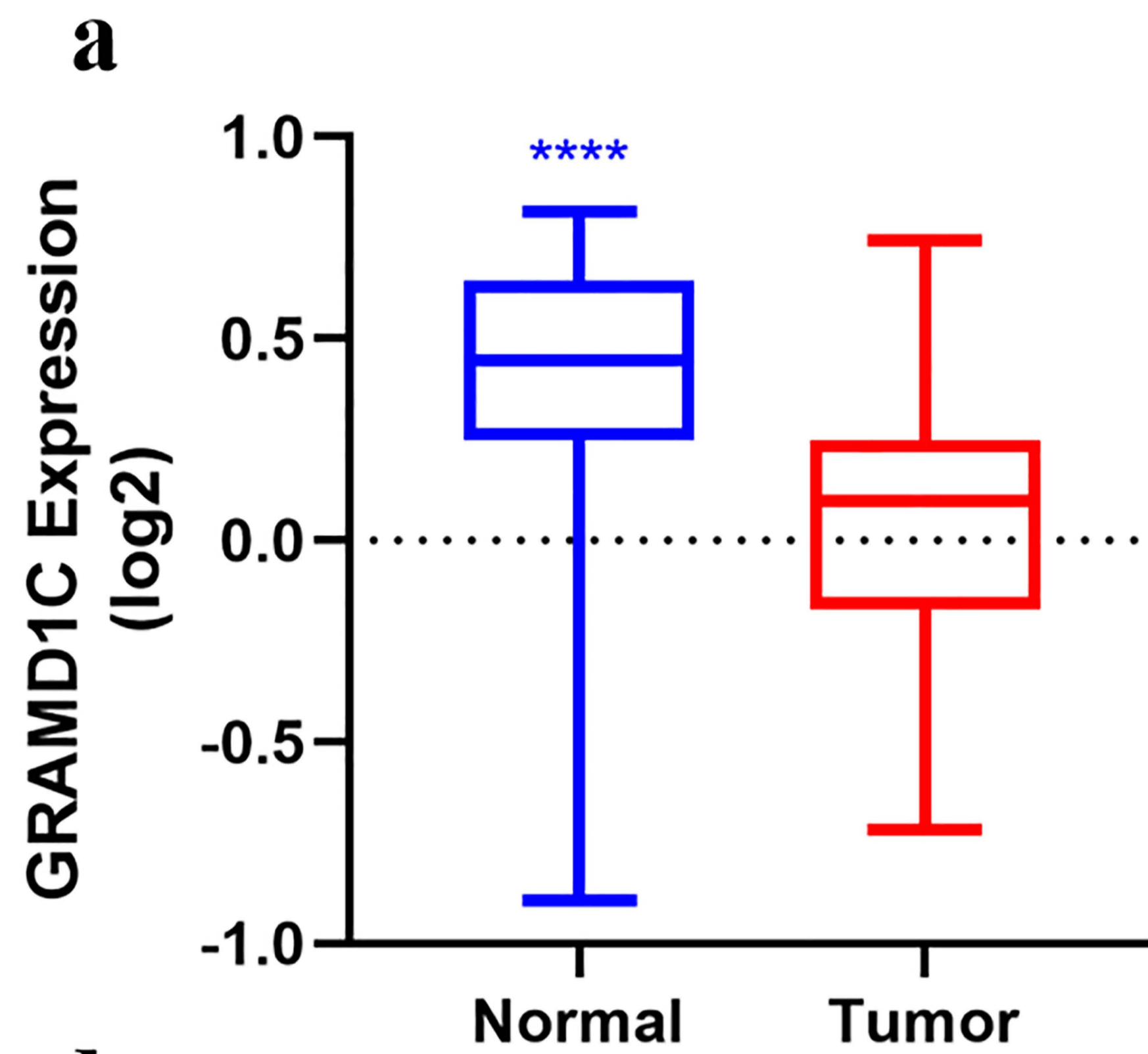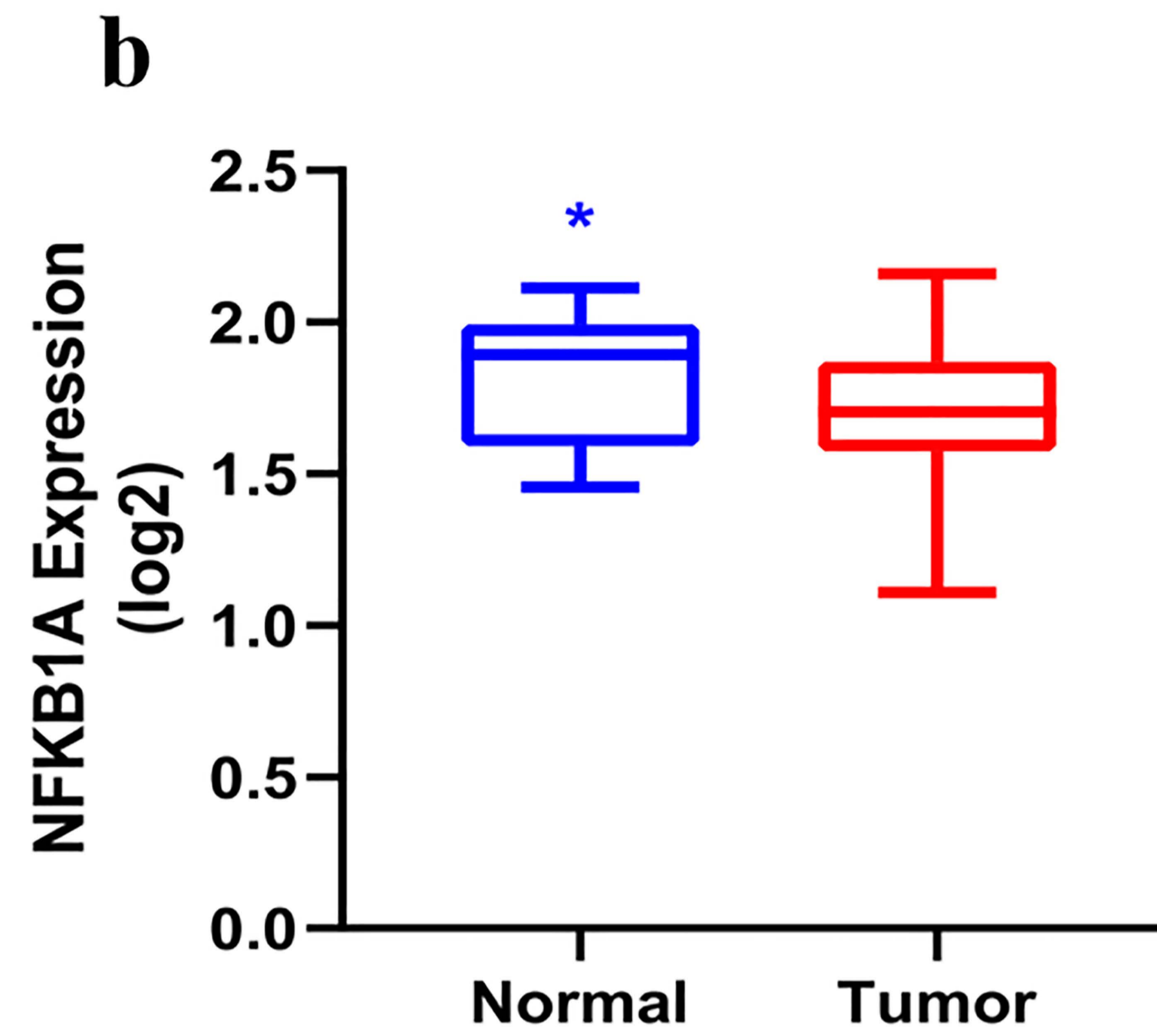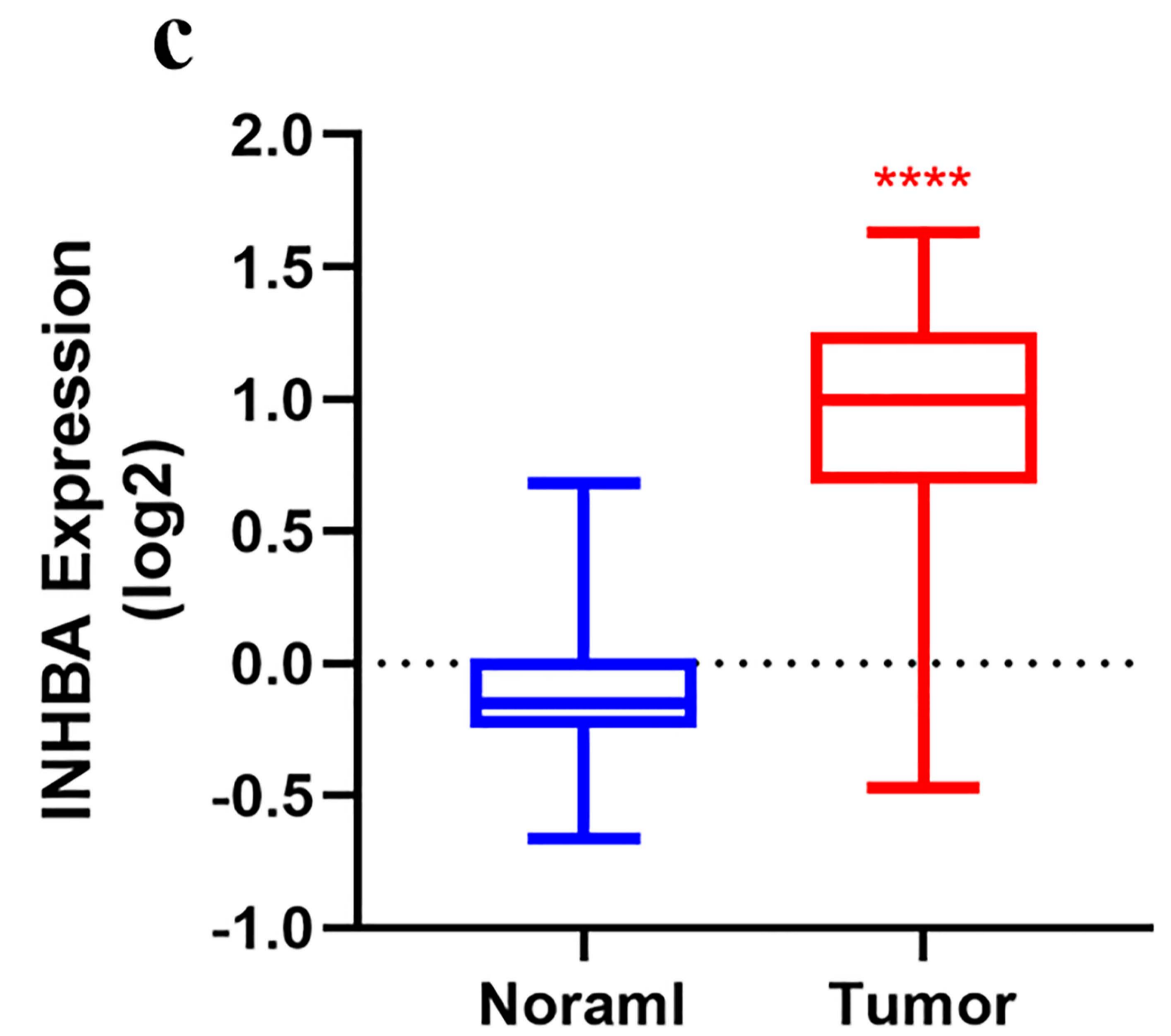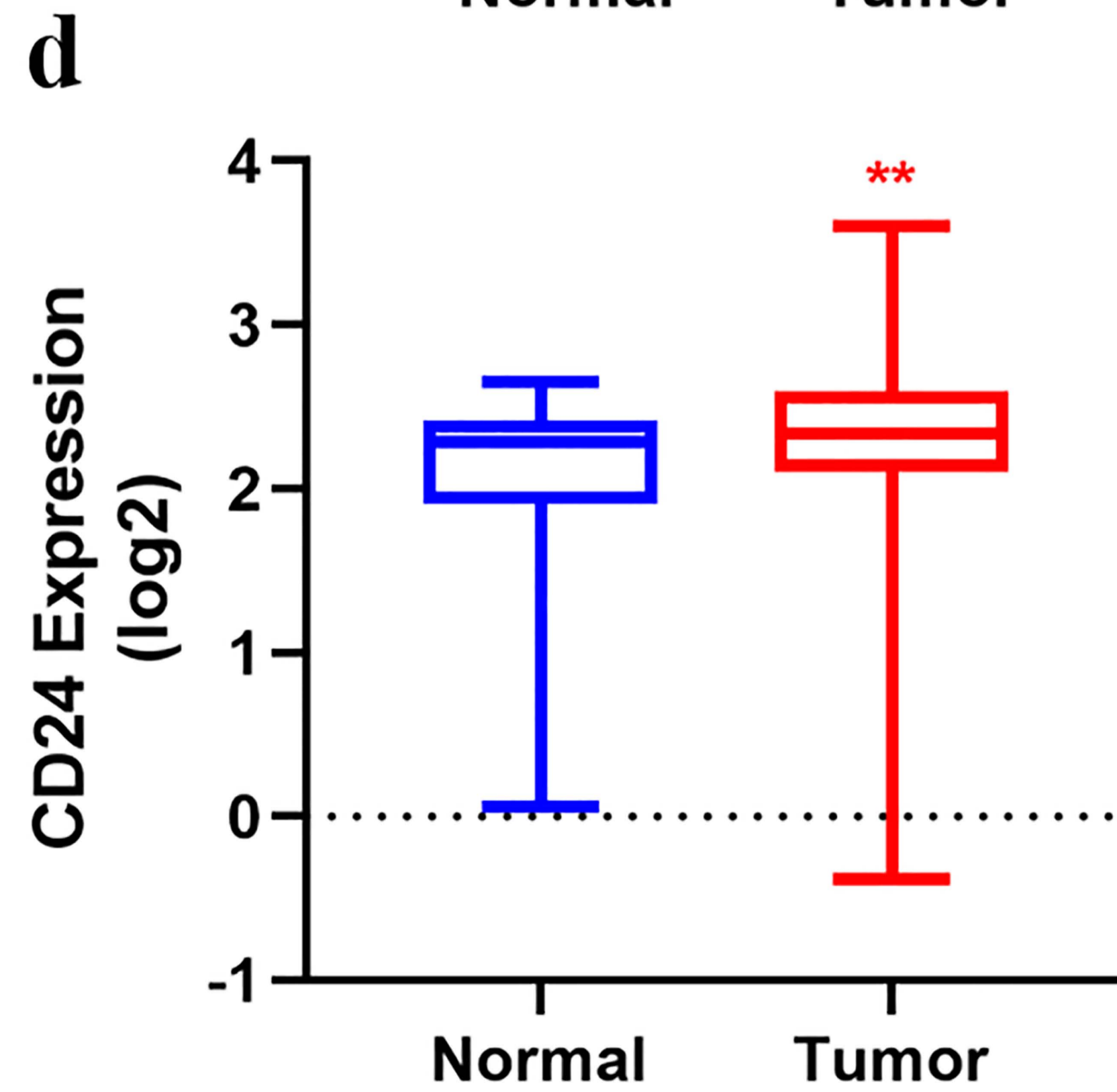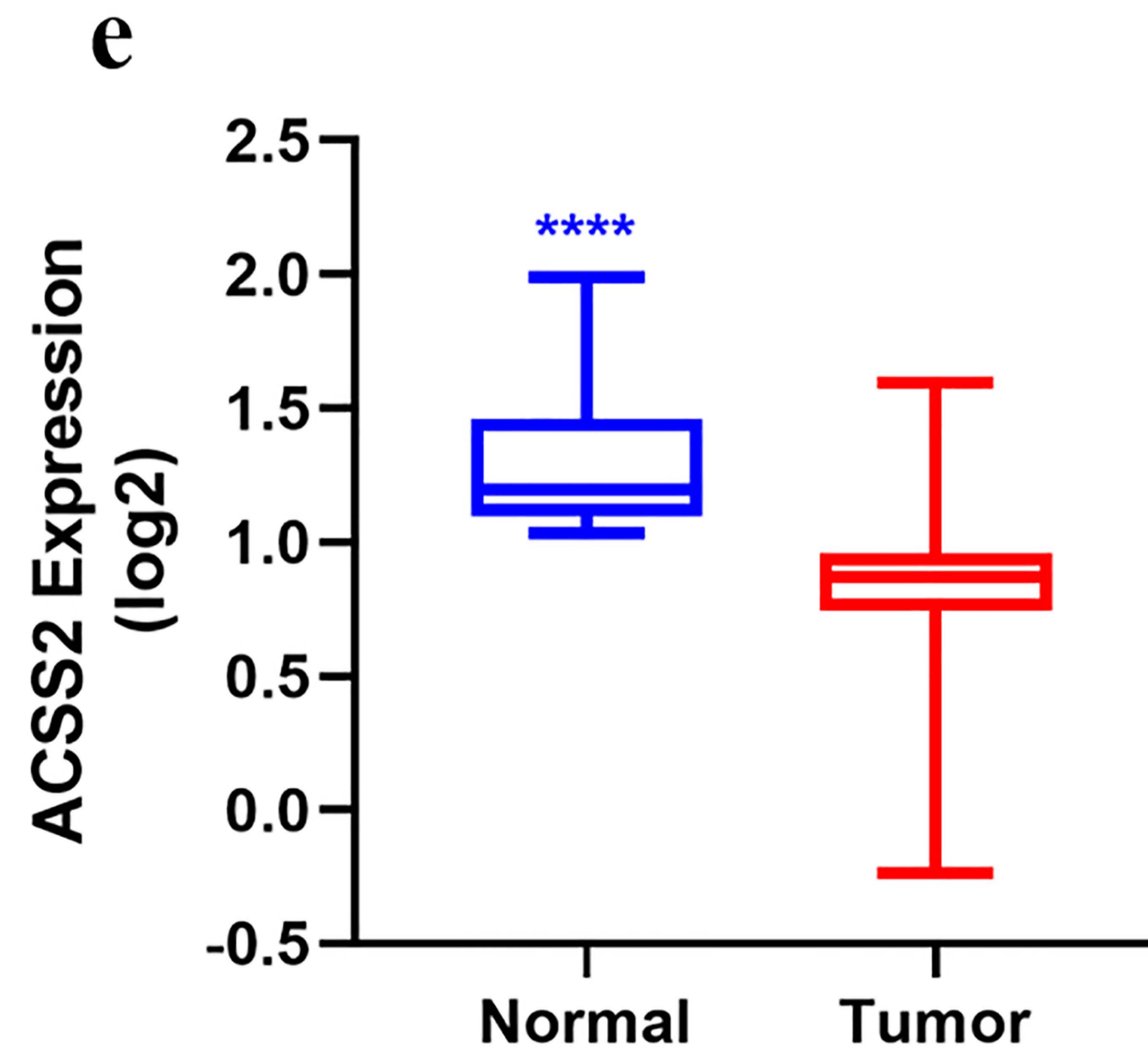

Supplement: Supplemental Information 3 — (A) GRAMD1C; (B) NFKBIA; (C) INHBA; (D) CD24; (E) ACSS2. The data are represented as the mean ±SD. * P = 0.0096, ** p = 0.0102 and *** p < 0.001. [file peerj-10-13922-s003.pdf]
